# A resting‐state study of volumetric and functional connectivity of the habenular nucleus in treatment‐resistant depression patients

**DOI:** 10.1002/brb3.1229

**Published:** 2019-02-25

**Authors:** Shu‐xin Luan, Lei Zhang, Rui Wang, Hua Zhao, Chang Liu

**Affiliations:** ^1^ Department of Mental Health The First Hospital of Jilin University Changchun China; ^2^ Department of Physiology, College of Basic Medical Sciences Jilin University Changchun China; ^3^ Department of Radiology The First Hospital of Jilin University Changchun China

**Keywords:** functional connectivity analysis, habenular nucleus, rs‐fMRI, treatment‐resistant depression

## Abstract

**Objective:**

To investigate the volumetric and functional connectivity of the habenular nucleus in treatment‐resistant depression (TRD) patients using the resting‐state functional magnetic resonance imaging (rs‐fMRI) approach.

**Methods:**

A total of 15 TRD patients, who visited the Mental Health Institute of the First Hospital Affiliated with Jilin University between August 2014 and March 2015, along with 15 normal subjects, were enrolled into this study for structural and functional imaging. Functional connectivity analysis was performed using bilateral habenular nuclei as the region of interest in contrast to whole‐brain voxels.

**Results:**

No significant difference of absolute volume was found in bilateral habenular nuclei between TRD patients and healthy controls, or after controlling for individual total intracranial volume. However, functional connectivity analysis showed increased connectivity between the right habenular nucleus with the medial superior frontal gyrus, anterior cingulate cortex and medial orbitofrontal gyrus, and decreased connectivity with the corpus callosum in the TRD group. For the left habenular nucleus seed, the brain region with increased functional connectivity in the inferior temporal gyrus and decreased functional connectivity in the insular was found in the TRD patients.

**Conclusion:**

Abnormal functional connectivity was present between the habenular nucleus and the default mode network in TRD patients. Dysfunction in habenular nucleus‐related circuitry for processing negative emotion might form the pathological basis for TRD. Significant asymmetric functional connectivity was also found between bilateral habenular nuclei in TRD patients. Such asymmetry suggests potentially divergent strategy for intervention on bilateral habenular nucleus regions in the future management of depression.

## 1 INTRODUCTION

Major depressive disorder (MDD) is a common psychiatric disorder with a protracted course, inflicting a major burden on the patient, the family and the society. The World Health Organization has estimated that MDD will become the second major disease burden, only after coronary heart disease (Lopez & Murray, 1998). Most MDD patients respond to pharmaceutical and psychological intervention, but still 20%–30% patients are refractory to various treatments (Peterson, Burgess, Dell, & Eberhard, 2001). In clinics, treatment‐resistant depression (TRD) is defined as those patients who fail to achieve satisfactory results with sufficient treatment by two or more antidepressant treatment approaches after the recent onset of depression (Nierenberg & Amsterdam, 1990). Due to frequent recurrences and unsatisfactory therapeutic efficiency of drugs, TRD has incurred severe economic and psychological burdens for patients, and is also a major therapeutic challenge for physicians. To optimize TRD treatment, knowledge of the pathogenesis of TRD has become increasingly important.

In recent years, resting‐state functional magnetic resonance imaging (rs‐fMRI) has become widely applied in studying brain functional changes (Rosazza & Minati, 2011). The functional connectivity approach has been used to reveal differential levels of blood‐oxygen dependency, and applied for the etiological study of psychiatric disorders (Kim & Lee, 2012). Increasing evidence from brain rs‐fMRI studies suggest that abnormal functional connectivity of the mesolimbic‐cortical pathway plays an important role in the pathogenesis of depression (Anand, Li, Wang, Lowe, & Dzemidzic, 2009; Frodl et al., 2009). The habenular nucleus, a pair of lateral nuclei located next to the third ventricle and posterior to the dorsal striatum, can be divided into the medial and lateral habenular nucleus (Andres, During, & Veh, 1999; Díaz, Bravo, Rojas, & Concha, 2011). Neuroanatomical study has revealed that the habenular nucleus mainly receives forebrain fiber projections originating from the cerebral cortex, basal ganglia, and lateral hypothalamus, and projects to the ventral tegmental area and substantia nigra compacta (Omelchenko, Bell, & Sesack, 2009), plus other limbic nuclei enriched with 5‐hydroxytryptamine (5‐HT) neurons including the dorsal and medial raphe (Araki, McGeer, & Kimura, 1988; Peyron, Petit, Rampon, Jouvet, & Luppi, 1998). Therefore, the habenular nucleus is one important relay station between the cortex and the limbic midbrain (Bianco & Wilson, 2009). It is widely involved in multiple physiological processes including sleep, reward, pain, sexual behavior, motor inhibition, and biorhythm (Lecourtier & Kelly, 2007). Due to its unique position, the role of the habenular nucleus in TRD has drawn intense research interests recently (Gass et al., 2014). Animal study has reported that pharmaceutical inhibition of the lateral habenular nucleus can improve depressive behaviors in TRD model (Winter, Vollmayr, Djodari‐Irani, Klein, & Sartorius, 2011). Similar results were obtained in humans as deep brain stimulation on the lateral habenular nucleus can cure persistent TRD (Sartorius et al., 2010).

However, little knowledge has been obtained regarding volumetric and functional connectivity of the habenular nucleus in MDD patients. Previous studies have revealed decreased volume and hyper‐activity of the habenular nucleus in bipolar disorder or post‐traumatic stress disorder patients by high‐resolution MRI (Savitz, Bonne et al., 2011; Savitz, Nugent et al., 2011). In contrast, Schmidt et al reported that absolute and relative total and hemispheric habenula volumes did not differ significantly between the unmedicated MDD patients, medicated MDD patients, and healthy control (Schmidt et al., 2017). Most recently, Schafer et al also found that habenula volume was not significantly different in a case–control study for the patients with schizophrenia, bipolar disorders, and demographically matched healthy individuals (Schafer et al., 2018). While limited studies have been done for functional connectivity of the brain, animal studies have demonstrated that significant asymmetry exists in structure, function, and morphology between the left and right habenular nucleus (Bianco & Wilson, 2009). Aim of the current study was, therefore, to conduct structural and rs‐fMRI studies in order to execute volumetric computation and functional connectivity analysis of bilateral habenular nuclei between TRD patients and normal control cohort, which might be used for the diagnosis and treatment of TRD.

## 2 SUBJECTS AND METHODS

### 2.1 Participants

Fifteen TRD patients, who visited the Mental Health Institute of the First Hospital affiliated to Jilin University between 2014 and 2015, were enrolled in this study. TRD was diagnosed by two experienced psychiatrists. Treatment resistance was defined as nonresponsiveness to at least two antidepressant courses with standard dosage and duration (at least 6 weeks for each treatment course) (Nierenberg & Amsterdam, 1990). Nonresponsiveness was defined as less than 50% reduction in the 17‐item Hamilton Rating Scale for Depression (HRSD) after treatment with a minimum dose of 60 mg/day of fluoxetine equivalents. All patients meet the following inclusion criteria: (a) during an ongoing major depressive episode diagnosed in accordance with Structured Clinical Interview for DSM‐IV (First, Spitzer, Gibbon, & Williams, 1996), (b) total depressive duration was more than 2 years, (c) 18–55 years of age, (d) right‐handed Han Chinese, and (e) a 17‐item HRSD score ≥24.

The control group consisted of 15 healthy individuals from Jilin University, including staffs and students. They were matched with the TRD group in age, gender, and education level. Individuals were excluded if they had any of the following: (a) history of neurological diseases, organic brain disorders, or other medical illnesses; (b) any other Axis‐I psychiatric disorders such as schizophrenia, bipolar disorders, anxiety disorders, alcohol, or drug dependence; (c) pregnancy; (d) any contraindication for MRI. The study was approved by the Ethics Committee of the authors’ affiliated hospital and was conducted in accordance with the Declaration of Helsinki. All participants had full knowledge of the procedures and provided written informed consents.

### 2.2 Image acquisition

MRI images were acquired on a 3.0 T GE Discovery MR750 (General Electric, USA) using a conventional eight‐channel quadrature head coil. All subjects were instructed to relax, remain still, keep eyes closed, stay awake, and clear their heads of all thoughts during imaging.

For high‐resolution anatomical scan, the three‐dimensional (3D) T1‐weighted scans were acquired with a spoiled gradient‐recalled acquisition in steady‐state (SPGR) sequence. Parameters were as following: TR = 8.2 ms, TE = 3.2 ms, slice thickness = 1.0 mm (no slice gap), voxel size (REC) = 0.5 × 0.5 × 1.0 mm^3^, field of view: 240 × 240 mm, acquisition matrix = 256 × 256, flip angle = 15°, NEX = 1,192 slices. For each participant, high‐resolution 3D T1‐weighted images were acquired for whole‐brain coverage, with a sagittal slice orientation.

For resting‐functional scan, the rs‐fMRI images were scanned using a T2*‐weighted gradient‐echo planar imaging sequence with the following parameters: TR = 2000 ms; TE = 30 ms, flip angle = 90°, voxel size = 3.75 × 3.75 × 3 mm (no slice gap), field of view = 240 × 240 mm, with 44 axial slices parallel to the AC‐PC line, and acquisition time = 6 min.

All the subjects did not complain any discomfort or feel asleep during the scan. No obvious structural damage was observed based on the conventional MR images.

### 2.3 Habenular nucleus seed segmentation

**Figure 1:**
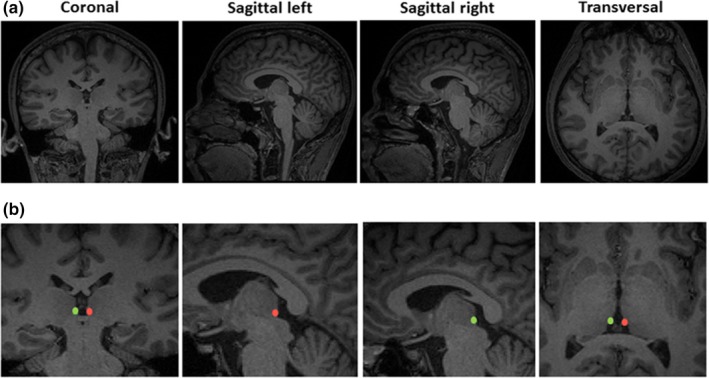
Images of the habenular nuclei. Habenular nuclei in the coronal, transversal, and sagittal planes. Native T1 maps (a) and T1 maps with manually segmented habenular nucleus (b). Right habenular nucleus voxel overlay in green and left habenular nucleus voxel overlay in red

Due to the small size of the habenular nucleus, we placed the region of interest (ROIs) of the left and right habenular nucleus on each participant's T1‐weighted structural image. The habenular nucleus was located in the epithalamus and appeared brighter than the surrounding cerebrospinal fluid (CSF) and gray matter. Both left and right habenular nucleus seeds were segmented manually by two independent clinicians in the tri‐planar view using ITK‐SNAP3.6.0 version 3.6.0 (Yushkevich et al., 2006) (Figure 1). The absolute volume of the left and right habenular nucleus was automatically calculated for each participant by using ITK‐SNAP3.6.0.

### 2.4 Preprocessing of functional imaging data

Functional MRI data preprocessing was performed using GRETNA software (Wang et al., 2015), which works with SPM12 (www.fil.ion.ucl.ac.uk/spm/software/spm12/) implemented on the MATLAB platform. Before preprocessing, volumes at the first 5 time points were discarded during the adaption phase for reaching a steady state. Then, after slice timing and head motion correction, spatial normalization to the Montreal Neurological Institute atlas space was conducted for each participant (2‐mm isotropic voxels). Notably, the ROIs of the habenular nucleus were masked out during spatial smoothing to ensure functional specificity. Subsequently, images were smoothed using a Gaussian kernel of 6 × 6×6 mm full‐width at half maximum. These images were processed by linear detrending and band‐pass filtering (0.01–0.08 Hz). Finally, white matter signal, CSF signal, and Friston 24 motion parameters were regressed out from the time series of each voxel (Friston, Williams, Howard, Frackowiak, & Turner, 1996).

### 2.5 Whole‐brain seed‐based functional connectivity analysis

To conduct habenular nucleus‐based functional connectivity analysis, individual ROIs of the habenular nucleus were first registered to the Montreal Neurological Institute space (2‐mm isotropic voxels) according to the deformation fields that were derived from tissue segmentation of each participant's T1 weighted image. All the ROIs of the habenular nucleus were carefully inspected; the mean time series of the left and right habenular nucleus was extracted and correlated with the time series of each voxel over the entire brain, thus generating two whole‐brain functional connectivity maps for each participant. Finally, a Fisher's r‐to‐z transformation was applied to all the functional connectivity maps to improve normality.

### 2.6 Statistical analysis

Two‐sample *t* test was used to compare differences of age, education level, and HRSD scores between TRD patients and healthy controls. rsFC analyses were carried out using the REST V1.8 package (Song et al., 2011). To explore brain regions showing significant functional connectivity to the left and right habenular nucleus within each group, one‐sample *t* test (*p* < 0.001, AlphaSim corrected) was performed on individual normalized rsFC maps from each group. Then, to explore differences in rsFC between TRD patients and healthy controls, a two‐sample *t* test was performed in a voxel‐by‐voxel manner, using age, gender, and gray matter volume as covariates to avoid any confounding effects. All results were presented at the significant level of *p < *0.001 using AlphaSim correction, and clusters that were greater than 13 voxels were applied to the resulting statistical map.

## 3 RESULTS

### 3.1 Demographic and baseline characteristics of the study subjects

Detailed clinical and demographic characteristics of patients are shown in Table 1.

**Table 1:** Demographic and baseline characteristics of the participants

Variables	TRD (*n = *15)	Controls (*n = *15)	*p* value	
Male gender, *n *(%)	9 (60.0)	7 (46.7)		0.715
Age, years				0.696
Mean (*SD*)	34.4 (6.2)	33.5 (6.8)		
Range	23–43	28–55		
Education, years				0.314
Mean (*SD*)	15.7 (1.9)	16.3 (1.6)		
Range	12–19	15–20		
HRSD scores				<0.001
Mean (*SD*)	27.5 (2.9)	5.9 (1.2)		
Range	25–35	3–7		
Duration of disease, years		—		—
Mean (*SD*)	6.9 (3.3)			
Range	2–13			

HRSD: Hamilton Rating Scale for Depression; TRD: treatment‐resistant depression.

TRD patients were comparable with healthy controls in gender, age, and education level (*p* > 0.05). TRD patients had significantly higher HRSD scores than healthy controls (*p < *0.05; Table 1).

### 3.2 Volumetric analysis of the habenular nucleus

Comparing the TRD patients and healthy control participants, there was no significant difference in absolute volume of the left (controls, 22.73 ± 1.87 mm^3^; TRD, 22.33 ± 3.06 mm^3^; *p = *0.304) and right habenular nuclei (controls, 18.73 ± 2.49 mm^3^; TRD, 18.87 ± 2.47mm^3^; *p* = 0.418). There was no significant difference in the total intracranial volume between the two groups (*p* = 0.123). After controlling for individual total intracranial volumes, no significant difference was found between the two groups in the volume of left (*p* = 0.759) and right (*p* = 0.735) habenular nuclei (Table 2).

**Table 2:** Habenular nuclei volumetric parameters (mm^3^)

Variables	TRD (*n = *15)	HC (*n = *15)	*p* value
Mean left absolute volume ± *SD*	22.33 ± 3.06	22.73 ± 1.87	0.670
Mean right absolute volume ± *SD*	18.73 ± 2.49	18.87 ± 2.47	0.884
Mean total intracranial volume ± *SD*	1,464.97 ± 122.13	1,519.56 ± 124.46	0.236
Mean left habenular nucleus ± *SD*	0.0153 ± 0.0024	0.0150 ± 0.0018	0.672
Mean right habenular nucleus ± *SD*	0.0129 ± 0.0019	0.012 ± 0.0018	0.295

TRD: treatment‐resistant depression; HC: healthy control.

### 3.3 Habenula resting‐state functional connectivity

**Figure 2:**
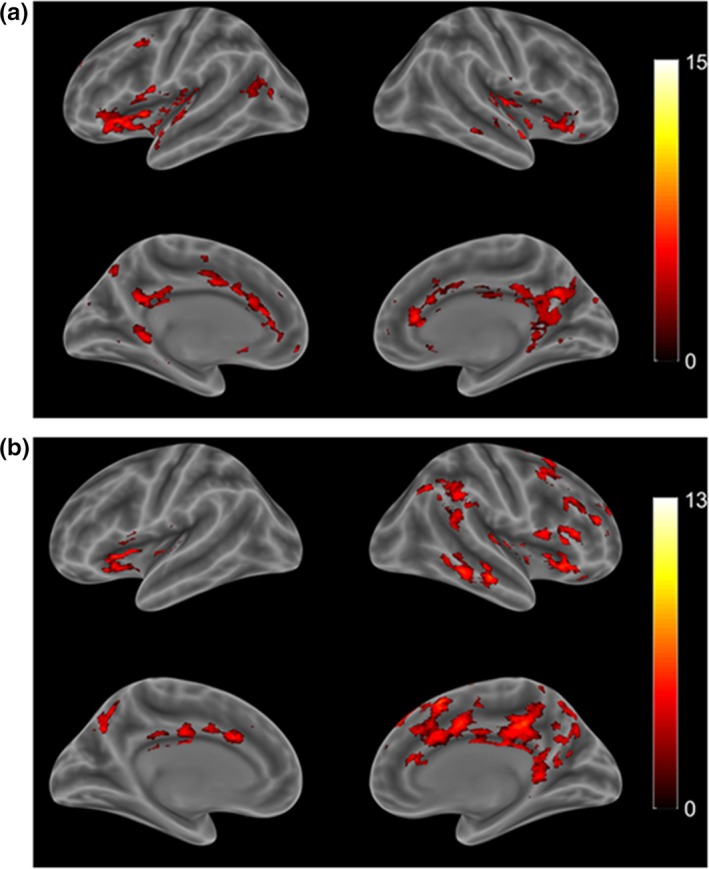
Functional connectivity with bilateral habenular nuclei in healthy subjects. (a) Left lateral view under rs‐fMRI; (b) Right lateral view. Red scale indicates positive to negative connectivity with the habenular nuclei under the resting state. (One‐sample *t* tests, with a *p < *0.001 threshold; Alphasim corrected, Cluster size >13 voxels). rs‐fMRI: resting‐state functional magnetic resonance imaging

Brain regions of healthy controls showed positive functional connectivity with the right habenular nucleus (Table 3); these regions included the inferior frontal gyrus, pallidum, insular, caudate, superior frontal gyrus, supplementary motor area, medial frontal gyrus, anterior cingulate gyrus, and the middle temporal gyrus. For the left habenular nucleus, regions with positive functional connectivity consisted of the insular, inferior frontal gyrus, thalamus, caudate, pallidum, insular, anterior cingulate cortex, middle cingulate cortex, lingual gyrus, and inferior temporal gyrus (Figure 2).

**Table 3:** Functional connectivity with the habenular nuclei in healthy controls

Brain regions	Side	Cluster size (voxels)	*t*‐value	Peak MNI coordinate (mm)		
				*x*	*y*	*z*
Right habenular nucleus‐whole brain						
Middle temporal gyrus	L	34	6.587	62	−38	−12
Insular	L	488	6.331	−32	16	−12
Inferior frontal gyrus	R	156	8.002	34	22	−6
Pallidum	L	74	6.871	−16	−4	0
Middle frontal gyrus	R	53	6.692	32	46	28
Anterior cingulate gyrus	R	232	8.084	4	20	36
Superior frontal gyrus	R	113	7.491	12	34	54
Supplementary motor area	R	57	8.552	10	14	54
Medial frontal gyrus	R	88	9.210	28	12	58
Caudate	R	288	6.892	12	7	16
Thalamus	L	3,447	13.780	8	−24	2
Left habenular nucleus‐whole brain						
Inferior temporal gyrus	L	48	7.905	−50	−52	−26
Inferior frontal gyrus	R	290	7.639	44	22	−12
Thalamus	L	1,343	13.041	10	−26	2
Caudate	L	211	6.892	−13	12	12
Pallidum	L	87	5.912	−20	−1	−7
Insular	L	90	8.384	−40	−16	14
Anterior cingulate cortex	R	161	7.326	4	36	14
Middle cingulate cortex	L	1,226	8.287	−2	14	36
Lingual gyrus	R	48	6.087	4	−78	−10
Insular	R	70	7.484	38	−26	14

MNI: Montreal Neurological Institute.

**Figure 3:**
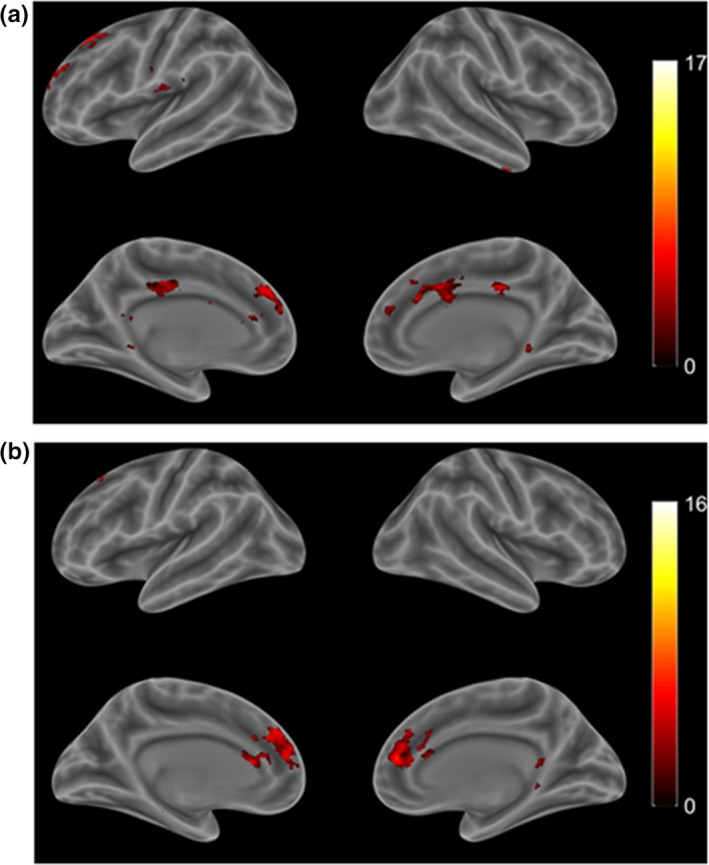
Functional connectivity with bilateral habenular nuclei in TRD patients. (a) Left lateral view under rs‐fMRI; (b) Right lateral view. Red scale indicates positive to negative connectivity with the habenular nuclei under the resting state. (One‐sample *t* tests, with a *p* < 0.001 threshold; Alphasim corrected, Cluster size >13 voxels). TRD: treatment‐resistant depression; rs‐fMRI: resting‐state functional magnetic resonance imaging

Treatment‐resistant depression patients exhibited positive functional connectivity between the right habenular nucleus with the anterior cingulate cortex, middle frontal gyrus, fusiform, pons, thalamus, medial orbitofrontal cortex, inferior temporal gyrus, insula, medial prefrontal cortex, and supplementary motor area (Table 4). The left habenular nucleus showed positive functional connectivity with the pons, anterior cingulate cortex, thalamus, ventral tegmental area, fusiform, superior frontal gyrus, interpeduncular nucleus, hippocampus, middle frontal gyrus, posterior cingulate cortex, medial prefrontal cortex, and caudate (Figure 3).

**Table 4:** Functional connectivity with the habenular nuclei in TRD patients

Brain Region	Side	Cluster size (voxels)	*t*‐value	Peak MNI coordinate (mm)		
				*x*	*y*	*z*
Right habenular nucleus‐whole brain						
Supplementary motor area	R	33	10.838	2	6	70
Middle frontal gyrus	L	71	5.441	−24	24	44
Fusiform	R	37	6.802	38	−14	−38
Pons	L	58	6.470	0	−32	−22
Thalamus	L/R	692	16.348	2	−24	2
Medial orbitofrontal cortex	L	68	8.595	−4	56	−4
Inferior temporal gyrus	R	70	7.965	54	−10	−30
Insular	R	26	5.602	34	22	8
Medial prefrontal cortex	R	686	7.159	12	42	14
Anterior cingulate cortex	L	27	8.206	−4	28	14
Left habenular nucleus‐whole brain						
Fusiform	R	105	9.095	40	−12	−36
Pons	R	32	5.691	0	−36	−26
Thalamus	L/R	1,042	17.662	4	−26	2
VTA	L	202	8.673	−6	−16	−14
Anterior cingulate cortex	L	126	5.480	−2	44	16
Inferior temporal gyrus	R	116	6.201	52	−12	−42
Precuneus	L	34	6.578	−8	−70	62
Caudate	L	139	5.319	−10	5	10
Anterior cingulate cortex	R	210	6.427	8	6	32
Hippocampus	L	59	5.673	−22	−30	8
Middle frontal gyrus	L	209	7.312	−22	24	44
Posterior cingulate cortex	R	100	5.742	6	−22	36
Medial prefrontal cortex	L	193	6.479	−10	42	34
Superior frontal gyrus	L	70	6.119	−22	42	28

TRD: treatment‐resistant depression; MNI: Montreal Neurological Institute; VTA: ventral tegmental area.

**Figure 4:**
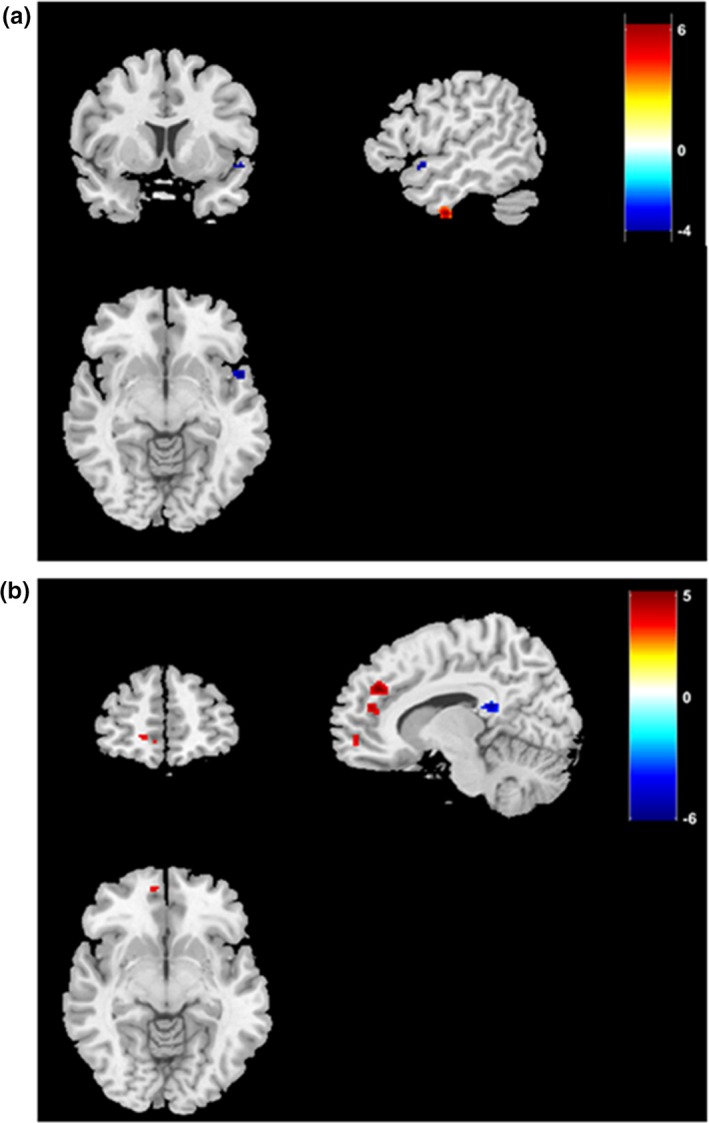
Functional connectivity toward the habenular nuclei between healthy subjects and TRD patients. Brain regions exhibiting increased (red) or decreased (blue) resting‐state functional connectivity with the left (a) and right (b) habenular nuclei in TRD subjects compared with healthy controls were shown in the coronal, sagittal, and axial views with the MNI location. The color bar indicates the *t‐*value. TRD: treatment‐resistant depression; MNI: Montreal Neurological Institute

We further compared functional connectivity between TRD patients and healthy controls, and found that toward the right habenular nucleus, several brain regions including the medial superior frontal gyrus, anterior cingulate cortex (ACC), medial orbitofrontal gyrus showed increased functional connectivity, whilst CC showed decreased functional connectivity (Table 5). In left habenular nucleus, the inferior temporal gyrus showed increased functional connectivity while the insular showed decreased functional connectivity (Figure 4).

**Table 5:** Comparison of functional connectivity toward the habenular nuclei between healthy controls and TRD patients

Brain regions	Laterality	Cluster size (voxels)	*t*‐value	Peak MNI coordinate (mm)		
				*x*	*y*	*z*
Right habenular nucleus						
TRD > controls						
Medial superior frontal gyrus	L	248	5.148	−10	40	32
Medial orbitofrontal Gyrus	L	77	4.405	−8	56	−4
Anterior cingulate cortex	L	36	3.697	−10	46	6
Controls > TRD						
Corpus callosum	L	60	−6.029	−10	−38	18
Left habenular nucleus						
TRD > controls						
Inferior temporal gyrus	R	74	6.682	52	−10	−42
Controls > TRD						
Insular	R	117	−4.459	50	8	−8

TRD: treatment‐resistant depression; MNI: Montreal Neurological Institute.

## 4 DISCUSSION

The volume of the habenular nucleus has been shown to be associated with MDD as suggested by postmortem examination (Ranft et al., 2010). For imaging study, due to the relatively small volume of the habenular nucleus (Savitz, Nugent et al., 2011), we chose a manual segmentation approach to study TRD patients in order to minimize bias. However, we still could not identify significant difference in the volume of the habenular nuclei in TRD patients. This was inconsistent with previous findings showing increased bilateral habenular nuclei volumes in untreated MDD patients with disease progression (Schmidt et al., 2017). Although Schmidt and colleagues reported that volumetric alteration of bilateral habenular nuclei was found only in unmedicated MDD patients, but not in the medicated MDD patients (Schmidt et al., 2017). TRD patients of the current study had been treated with at least two kinds of antidepression medication. Therefore, the application of 5‐HT reuptake inhibitors in the patients of the current study might be associated with the results of nonsignificant volumetric alteration of the habenular nuclei because these drugs might help improve neural plasticity through stimulating brain‐derived neurotrophic factor (Daszuta, Ban, Soumier, Hery, & Mocaer, 2005; Dayer, 2014).

Besides volumetric analysis, we also studied whole‐brain functional connectivity using bilateral habenular nuclei as ROIs. Similar to previous findings in healthy controls (Ely et al., 2016; Erpelding et al., 2014), we found that bilateral habenular nuclei exhibited positive functional connectivity with the thalamus, anterior cingulate cortex, inferior frontal gyrus, insular, and left pallidum and ipsilateral caudate. The left pallidum and ipsilateral caudate are important nuclei of that basal ganglia that have direct projection with the habenular nucleus and forming an important part of reward circuitry (Bromberg‐Martin, Matsumoto, Hong, & Hikosaka, 2010; Hikosaka, Sesack, Lecourtier, & Shepard, 2008; Hong & Hikosaka, 2008). These results were largely consistent with those seen in normal people, in which the habenular nuclei showed enhanced connectivity with the forebrain and midbrain regions, indicating a role of the habenular nuclei in transmitting information between the cortex and the limbic system. However, we did not find functional connectivity between the habenular nucleus and the pons or ventral tegmental area, which are centers for 5‐HT and dopamine and closely related with depression, in the healthy subjects. This might be due to the inhibition of the habenular nuclei on these regions under normal condition but not in the depressive state.

Previous studies have established the role of the habenular nuclei in depression. Metabolic rate of the habenular nuclei was enhanced in a model of learned helplessness in animals (Shumake, Edwards, & Gonzalez‐Lima, 2003) or under serotonin depletion (Morris, Smith, Cowen, Friston, & Dolan, 1999; Roiser et al., 2009). In the current study, TRD patients showed whole‐brain positive connectivity of bilateral habenular nuclei, implicating hyper‐activity of the habenular nuclei under depression. Previous studies reported that, compared to healthy controls, TRD patients showed positive connectivity between the habenular nuclei and the ipsilateral pons region, in which the raphe and locus coeruleus showed prominent connection with the habenular nuclei (Gottesfeld, 1983; Yang, Hu, Xia, Zhang, & Zhao, 2008). Therefore, enhanced habenular nuclei activity could decrease norepinephrine and 5‐HT release by inhibiting these two nuclei, and could be closely related with MDD pathogenesis (Chandley & Ordway, 2012; Zhao, Zhang, Yang, & Rusak, 2015). In addition, we observed positive connectivity between the left habenular nucleus and the ventral tegmental area, which is closely related with reward and negative emotion (Friedman, Friedman, Dremencov, & Yadid, 2008; Matsumoto, 2009). Animal studies showed direct fiber projection between the habenular nuclei and the ventral tegmental area and the rostromedial tegmental nucleus (Balcita‐Pedicino, Omelchenko, Bell, & Sesack, 2011; Root, Mejias‐Aponte, Qi, & Morales, 2014). Left habenular nucleus stimulation may inhibit dopaminergic neurons of the ventral tegmental area via stimulating GABAergic neurons in the rostromedial tegmental nucleus (Lavezzi, Parsley, & Zahm, 2012; Poller et al., 2011). The electrical stimulation for inhibiting the left habenular nucleus to ventral tegmental area projection could enhance dopaminergic activity for improving learned helplessness in rats (Li et al., 2011). The fMRI study also showed hyper‐connectivity between the ventral tegmental area and the left habenular nucleus when processing aversive events (Hennigan, D'Ardenne, & McClure, 2015), which was consistent with findings of the current study, that is, more brain regions presenting connection with the habenular nuclei in TRD patients in association with hyper‐activity of the habenular nuclei. A comparison between two sides showed more connectivity of the left habenular nucleus with depression‐related regions, including the ventral tegmental area, hippocampus, and posterior cingulate cortex. This was consistent with previous study showing higher activity of the left habenular nucleus when processing negative information (Kim & Lee, 2012). These data support asymmetrical function of the bilateral habenular nuclei.

Retrograde labeling study showed that the habenular nuclei received inputs from the anterior cingulate cortex, the prefrontal cortex, and the insular cortex (Vadovicova, 2014), which was consistent with DTI study showing the involvement of the habenular nuclei within an adversity circuit (Iwabuchi et al., 2014). It is interesting that such adversity circuit also consists of regions showing abnormal connectivity with bilateral habenular nuclei such as the medial superior frontal gyrus, anterior cingulate cortex, medial orbitofrontal gyrus, and insular, which participate in emotion and cognitive integration (Buckner, Andrews‐Hanna, & Schacter, 2008; Etkin, Egner, & Kalisch, 2011). These results indicated that dysfunction of adversity circuit containing the habenular nuclei might be at least partially implicated in TRD.

Our results also showed increased functional connectivity between the left habenular nucleus and the inferior temporal gyrus. Intergroup comparison indicated that the inferior temporal gyrus, cingulate cortex, prefrontal, and insular lobe are important components of the default model network in addition to the corpus callosum (Chen, Wang, Zhu, Tan, & Zhong, 2015). Increasing evidence focused on the correlation between the default model network and depression, and considered that dysfunction of the default model network plays crucial roles in the pathogenesis of depression (Hamilton, Farmer, Fogelman, & Gotlib, 2015; Li et al., 2013; Orosz et al., 2012). Meanwhile, functional connectivity between the default model network and other brain structures are also drawing attentions. Hamilton *et al.* found important effect of functional connectivity between the frontal cortex and the default model network in progression of depression (de Kwaasteniet et al., 2015). Another clinical study found weakened functional connectivity between the default model network and the cognitive control network in TRD patients (Bianco, Carl, Russell, Clarke, & Wilson, 2008). In our study, it was found that those regions with abnormal connectivity between the left and right focused on the default model network regions in TRD patients, further indicating that TRD pathogenesis may be closely related to abnormal functional connectivity between the habenular nuclei and the default model network.

Meanwhile, completely different functional connectivity maps were found between the two sides, indicating asymmetrical function of the habenular nuclei. Although the mechanism is still obscure, laterality bias of structure and function is one innate feature of the central nervous system (Concha & Wilson, 2001). Such feature can be raised at the early evolutionary phase and is closely related with personalized emotion and behavior (Aizawa, 2013; Concha, Signore, & Colombo, 2009). For example, zebra fish habenular nuclei showed asymmetry regarding forebrain afferent fibers or midbrain‐projecting fibers (Hendricks & Jesuthasan, 2007; Turner et al., 2016). A recent human study also showed asymmetrical functional connectivity between bilateral habenular nuclei using high‐resolution MRI (Hétu et al., 2016). Our results that TRD patients presented prominent asymmetrical functional connectivity may indicate different roles of bilateral habenular nuclei in MDD pathogenesis, although such difference requires further delineation, especially in humans. Therefore, different strategies may be required on both sides, such as high frequency stimulation on the left dorsolateral prefrontal cortex plus low‐frequency stimulus on the right side using the transcranial magnetic stimulation approach for antidepression (Chen et al., 2013).

To our knowledge, this is the first piece of data showing volumetric and functional connectivity on bilateral habenular nuclei in TRD patients. Some weakness and limitations, however, still exist such as the small cohort size due to more stringent inclusion criteria, plus the undifferentiating patterns between the medial and lateral habenular nuclei, which have major structural and functional deviations (Geisler & Trimble, 2008; Viswanath, Carter, Baldwin, Molfese, & Salas, 2013). In this regard, we plan to continue enrollment of TRD patients and further study the potential connection of volumetric and functional connectivity with bilateral habenular nuclei in TRD patients. In addition, our TRD patients did not receive drug‐free period before imaging out of ethical considerations; we thus should take antidepressant drugs into account when analyzing functional connectivity differences.

In summary, our results suggested that abnormal functional connectivity exists between the habenula nuclei and the default mode network in TRD patients and adversity processing circuit dysfunction is potentially involved in TRD pathogenesis. The prominently asymmetrical connectivity between bilateral habenular nuclei in TRD patients implies different strategy for bilateral habenular nuclei in antidepressant treatment.

## CONFLICT OF INTEREST

None declared.
